# On the Network Transmission Mechanisms of Disease-Specific Healthcare Expenditure Spillovers: Evidence from the Connectedness Network Analyses

**DOI:** 10.3390/healthcare9030319

**Published:** 2021-03-13

**Authors:** Wen-Yi Chen

**Affiliations:** Department of Senior Citizen Service Management, National Taichung University of Science and Technology, Taichung 40343, Taiwan; chenwen@nutc.edu.tw; Tel.: +886-4-22196932

**Keywords:** connectedness network analysis, connectedness index, disease-specific healthcare expenditure, spillovers, robust least square

## Abstract

Previous studies investigating factors influencing healthcare expenditure growth ignored the network transmission mechanisms of disease-specific healthcare expenditure spillovers and regarded the processes culminating in healthcare expenditure growth as a black box. In this study, we investigated factors influencing the network transmission mechanisms underlying the determinants of healthcare expenditure growth through the dynamic connectedness network and the robust least square regression analyses. Our results indicate that demographic transition and business cycles are key factors increasing interconnectedness of different disease-specific healthcare expenditures, and that promotion of primary care utilization would reduce total healthcare expenditure spillovers. In order to reduce diffusion of disease-specific healthcare expenditures, health promotion activities should focus on those clinical diagnosis-related groups of diseases classified as pure net transmitters of spillover, and preventive interventions targeting different diseases should be activated in different phrases of the business cycle.

## 1. Introduction

The persistent increase in healthcare expenditure is a potential risk jeopardizing the sustainability of the healthcare system and its capacity to deliver affordable healthcare services to the public. The major determinants of healthcare expenditure growth identified by previous studies include population ageing [1,2,3,4,5,6,7], business cycles and income [2,3,6,8,9,10,11,12], technological innovation [1,2,11,13,14,15], and Baumol’s cost disease, a term used to describe the increase of healthcare price due to unbalanced growth of productivities between the healthcare sector and the whole economy [3,16,17,18]. It is crucial to point out that the network transmission mechanisms underlying these determinants effecting healthcare expenditure change come from the expansion or compression of morbidities resulting from demographic, socioeconomic, and environmental changes in the process of human development [19,20]. Nevertheless, most of the previous studies investigating factors influencing healthcare expenditure growth ignored the network transmission mechanisms of disease-specific healthcare expenditure spillovers and regarded the processes culminating in healthcare expenditure growth as a black box. Therefore, the purpose of this study is twofold: First, we unravel the black box of the processes underlying healthcare expenditure growth by measuring disease-specific healthcare expenditure spillovers using the dynamic connectedness network analyses proposed by Antonakakis and his colleagues [21]. Second, we estimated the robust least square (RLS) regression model proposed by Yohai [22] to identify the network transmission mechanisms of disease-specific and total healthcare expenditure spillovers.

In fact, the increased interest in network analyses in the fields of social science and medicine is motivated by a movement by healthcare providers towards greater patient-sharing and care coordination [23,24]. From the perspective of medical cost control of various clinical diagnosis-related groups of diseases, the identification of dynamic connectedness network relationships among different disease-specific healthcare expenditures enables us to understand the directional diffusion of disease-specific healthcare expenditure and their aggregate total healthcare expenditure spillovers across a period of time. Nevertheless, it is essential to note that there are several challenges inherent in exploring the network transmission mechanisms underlying the determinants of healthcare expenditure growth. First, it is difficult to obtain consistently and sufficiently long time series data to allow effective examination of disease-specific morbidities and their healthcare expenditures. This is because the International Classification of Diseases (ICD) codes (an internationally consistent classification method used to classify different diseases) did not obtain popularity worldwide until early 1990, when the World Health Organization started to regularly announce consistent ICD codes and promoted international compatibility in healthcare data collection and reporting for the purposes of worldwide epidemiological surveillance and health management [25]. Second, healthcare expenditures related to different diseases may be jointly influenced by some unobserved factors such as comorbidity of and complications between diseases. Third, changes in some unobservable components of human development variables (such as greater preference for healthcare services due to increased longevity, elevated expectations for future income, hazard exposure predisposing the general public to diseases, etc.) would lead to a structural change in the data generating processes underlying disease-specific and total healthcare spillovers. Failure to accommodate these challenges would create biases when inferring connectedness network relationships among different disease-specific healthcare expenditures and obscure the network transmission mechanisms underlying disease-specific and total healthcare expenditure spillovers.

In response to the challenges of limited and inconsistent disease-specific time series data for disease-specific morbidity, Taiwan was selected as the target country under study. This is because expansion or compression of morbidity is likely to result from increased longevity, and Taiwan has experienced a rapid demographic transition (within 25 years) from an aging society to an aged society, and it was predicted to become a hyper-aged society by 2026 [26]. In addition, the Taiwanese healthcare system belongs to the National Health Insurance (NHI) system (characterized by a universal coverage program), and the government has administrated a population-level database (namely, the National Insurance Research Database) collecting consistent disease-specific time series data since 1995 intended to capture real-world evidence needed to support decisions on clinical treatments, community interventions and health policy-making. Moreover, Taiwan also underwent an extraordinary phase of economic development during the latter half of the 20th century. In 1960, the GDP per capita in Taiwan was only US $163, jumping to US $25,909 in 2019 [27]. This striking economic growth and rapid industrialization earned Taiwan the distinction of being known as one of the Four Little Asian Dragons (the title given to four cutting edge and high-paying economies) alongside Hong Kong, South Korea, and Singapore [28]. These features of having both dramatic demographic and economic transitions as well as a well-established healthcare system make Taiwan a suitable target country for our research.

Methodologically, we employed the dynamic connectedness network analyses, developed by Antonakakis and his colleagues [21], to establish dynamic connectedness network relationships among different disease-specific healthcare expenditures. In this way, the dynamic connectedness indices for disease-specific and total healthcare expenditure spillovers could be estimated to measure directional diffusion of disease-specific healthcare expenditures and their aggregate total healthcare expenditure spillovers. Note that the procedure for establishing dynamic connectedness networks used in this study is rooted in the forecast variance decomposition from the time-varying parameter vector autoregressive (TVP-VAR) model [21]. Several appealing features of TVP-VAR-based connectedness network analyses are worth highlighting: First, conventional dynamic connectedness network analyses are based on rolling-window estimation [29,30]. Because of this, the selection of rolling-window size and loss of observations involved within the rolling-window estimation process become two major issues in conventional dynamic connectedness network analyses. Instead of using conventional dynamic connectedness network analyses, we applied the TVP-VAR model suggested by Antonakakis and his colleagues [21] for dynamic connectedness network estimation, allowing us to prevent the potential issues common to conventional dynamic connectedness network analyses. Second, the vector autoregressive (VAR) model treats all variables in the model as endogenous variables, and the time-varying parameter can capture potential structural change in the data-generating process. Hence, the TVP-VAR estimation under the dynamic connectedness network analyses can simultaneously deal with structural change and endogeneity of different disease-specific healthcare expenditures, which is advantageous since these are two major challenges in the investigation of network transmission mechanisms underlying the determinants of healthcare expenditure growth.

This research contributes to the existing literature on the study of the determinants of healthcare expenditure growth in three ways: First, in contrast to previous research on the determinants of healthcare expenditure growth, this study elucidates the network transmission mechanisms underlying the determinants of healthcare expenditure growth rather than simply investigating factors influencing healthcare expenditure [1,2,3,4,5,6,7,8,9,10,11,12,13,14,15,16,17,18]. Second, we applied the TVP-VAR-based connectedness network analyses developed by Antonakakis and his colleagues [21] to construct dynamic connectedness indices for disease-specific and total healthcare expenditure spillovers. This allowed us to shed light on the block box of healthcare expenditure growth processes resulting from the expansion or compression of morbidities. Third, in order to better understand the network transmission mechanisms of disease-specific and total healthcare expenditure spillovers, we applied the RLS regression model introduced by Yohai [22] to identify a resistant relationship between the dynamic connectedness indices and various factors (such as demographic transition, business cycles, medical price, primary care utilization, Baumol’s cost disease, etc.) that affect the growth of healthcare expenditure. The results generated from this study provide reliable information on the network transmission mechanisms of disease-specific and total healthcare expenditure spillovers.

## 2. Materials and Methods

The determinants of disease-specific spillovers (measured by the net total directional connectedness index, *NTDCI_it_*) and total healthcare expenditure spillovers (indicated by the total connectedness index, *TCI_t_*) can be specified as follows:

(1) $$CI_{t}^{}=\pi_{0}+\pi_{1}DV_{t}+\pi_{2}SEV_{t}+\pi_{3}HU_{t}+\xi_{t}$$

where *CI_t_* denotes either *NTDCI_it_* or *TCI_t_*. *DV_t_*, *SEV_t_*, and *HU_t_* represent demographic variables (such as young-age and old-age economic dependency ratios), socio-economic variables (such as composite leading index, medical price index, and Baumol’s cost disease), and healthcare utilization (such as volume of primary care utilization), respectively. *π_i_* (*i* = 0, 1, 2, 3) are parameters we need to estimate and *ξ_t_* is the error term. Equation (1) was estimated by the RLS method [22] in order to obtain resistant results in the occurrence of outliers.

Note that the *TCI_t_* represents interconnectedness of the network of all different disease-specific healthcare expenditures. *NTDCI_it_* denotes the difference between total directional connectedness to others and from others for the disease-specific healthcare expenditure *i*. A positive sign for the *NTDCI_it_* demonstrates one condition, in which disease-specific healthcare expenditure *i* is driving the network, and a negative sign for the *NTDCI_it_* illustrates the other condition, in which disease-specific healthcare expenditure *i* is driven by the network. Finally, the net pairwise directional connectedness index (*NPDCI_ij_*) can be broken down by the *NTDCI_it_* to measure the variance of the overall shocks that the disease-specific healthcare expenditure *i* transmitted to the disease-specific healthcare expenditure *j* (i.e., the bidirectional relationship between healthcare expenditures of disease *i* and disease *j*). Please see the online Supplementary Materials for technical details.

The data used for the connectedness network analyses include healthcare expenditures for eighteen clinical diagnosis-related groups of diseases. These eighteen clinical diagnosis-related groups of diseases were classified using the multi-level clinical classifications software (CCS) categories from the US Agency for Healthcare Research and Quality (AHRQ) [31]. We retrieved weekly aggregate healthcare expenditure time series from the National Insurance Research Database in Taiwan for the period of 1 January 2000 to 30 September 2015, generating 822 weekly observations. This study period was chosen in response to consistency in the ICD 9 codes used in the CCS from the US AHRQ [31]. These data were converted to real healthcare expenditure per capita by dividing by the number of total population and deflated by the medical price index, setting the year of 2014 as the base period. These eighteen disease-specific healthcare expenditures were proved to be stationary time series data according to the PP (Phillips and Perron) unit root test.

Furthermore, *TCI* and *NTDCI* (hereafter, subscripts were skipped for brevity) served as the dependent variables for the RLS regression analyses. Note that the explanatory variables for the RLS regression analyses (such as young-age and old-age economic dependency ratios, composite leading index, medical price index, volume of primary care, and Baumol’s cost disease) belong to monthly time series data. For this reason, the weekly data of *TCI* and *NTDCI* were converted into monthly data by weekly average. In addition, we retrieved the Taiwanese age distribution, labor force, index of average regular earnings of employees in the healthcare sector (used to measure permanent salary income), share of employed laborers in the healthcare sector, medical price index, and industrial production index (as a proxy to measure the productivity of Taiwan’s economy) from the Macroeconomic Statistics and the Demographic Statistics Databases administrated by the Taiwan government. These data enabled us to calculate the young-age (old-age) economic dependency ratios (defined as the population aged 15 and less (aged 65 and above) divided by labor force), and Baumol’s cost disease (given by the difference between the changes in wage in the healthcare sector and productivity of Taiwan’s economy divided by the share of labors employed in the healthcare sector). This adjusted type of Baumol’s cost disease was suggested by Colombier [17]. Finally, the composite leading index (used to measure business cycles) and volume of primary care utilization (defined as the share of outpatient care visits provided by local clinics) were obtained from the Taiwan Business Indicators Database and National Insurance Research Database in Taiwan, respectively. The stationarity of these time series data were obtained either through extracting the cyclic components of these time series data by the Hodrick and Prescott filter method or taking the difference of time series [13,32,33,34]. The detailed results of unit root tests and the connectedness network analyses can be found in the online Supplementary Materials.

## 3. Results

**Figure 1:**
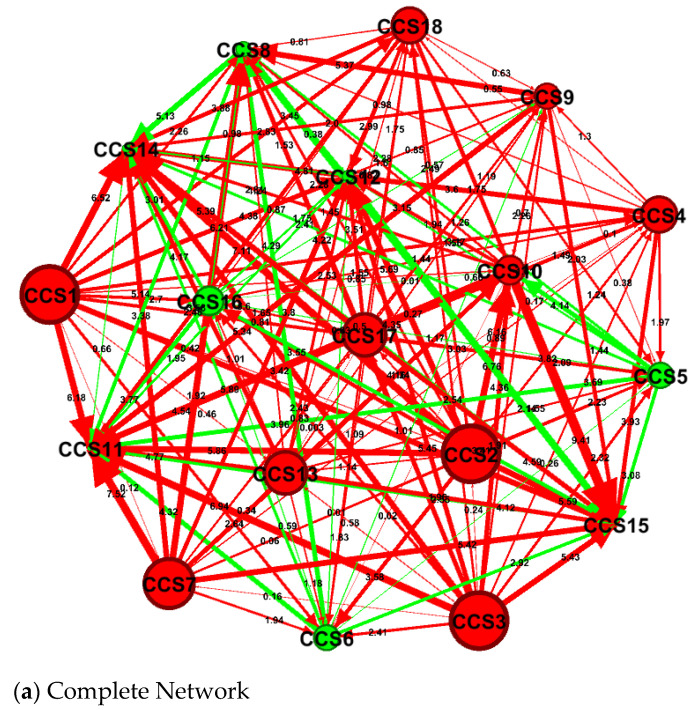

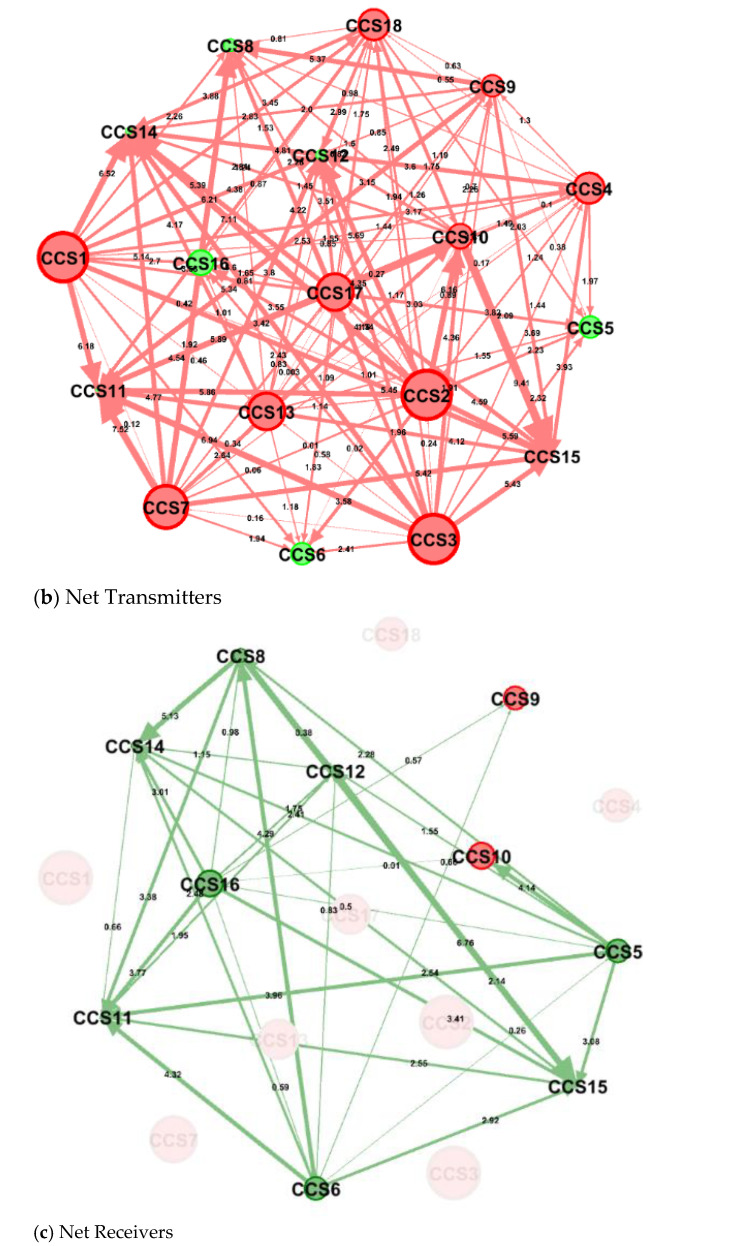
Static connectedness network structure of net-pairwise directional connectedness indices (*NPDCI*). (**a**) Complete Network: total connectedness index (*TCI*) is 87.20%. The size of nodes indicates the overall magnitude of transmission/reception of spillover for each clinical classification system (CCS) code. Red (green) color for a node denotes a specific CCS*i* (*i* = 1, 2,…, 18) that is a net transmitter (receiver). The thickness of arrows reflects the strength of the spillover between a pair of CCS codes. Thicker arrows indicate stronger spillovers between two CCS codes. (**b**) Net Transmitters: Ten of these eighteen clinical diagnosis-related groups of diseases (these being infectious and parasitic diseases (CCS1), neoplasms (CCS2), endocrine, nutritional, and metabolic diseases and immunity disorders (CCS3), diseases of the blood and blood-forming organs (CCS4), diseases of the circulatory system (CCS7), diseases of the digestive system (CCS9), diseases of the genitourinary system (CCS10) diseases of the musculoskeletal system and connective tissue (CCS13), symptoms, signs, and ill-defined conditions and factors influencing health status (CCS17), and residual codes unclassified diseases (CCS18) are net transmitters of spillover. (**c**) Net Receivers: The other eight of these eighteen clinical diagnosis-related groups of diseases (these being mental illness (CCS5), diseases of the nervous system and sense organs (CCS6), diseases of the respiratory system (CCS8), complications of pregnancy, childbirth, and the puerperium (CCS11), diseases of the skin and subcutaneous tissue (CCS12), congenital anomalies (CCS14), certain conditions originating in the perinatal period (CCS15), and injury and poisoning (CCS16)) are net receivers of spillover.

**Figure 2:**
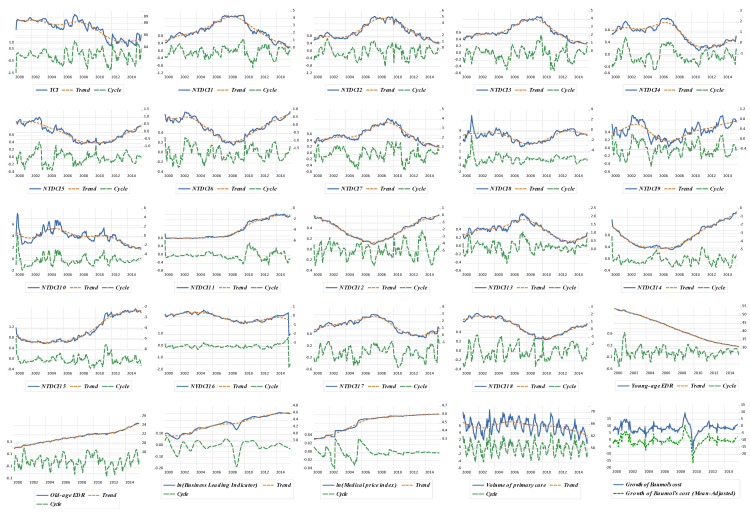
Time plots for variables used for the robust least square estimation. Note: *TCI* and *NTDCI_i_* (*i* = 1, 2,…, 18) represent total connectedness and net total directional connectedness indices of CCS code *i*, respectively. The monthly indices were aggregated from weekly indices. ln(●) is the nature logarithm operator. All trends were generated using the Hodrick–Prescott filter with a smoothing parameter (lambda = 14400). *X*-axis illustrates time horizon. Right *Y*-axis and left *Y*-axis indicate the trends of variables and their cyclical components, respectively. We could further separate these eighteen disease-specific healthcare expenditures into three groups: the first group being the pure net transmitters of spillover (all having positive signs of *NTDCI*), which includes the infectious and parasitic diseases (CCS1), neoplasms (CCS2), endocrine, nutritional, and metabolic diseases and immunity disorders (CCS3), diseases of the circulatory system (CCS7), and diseases of the musculoskeletal system and connective tissue (CCS13). The second group is the pure net receivers of spillover (all with negative signs of *NTDCI*), consisting of complications of pregnancy, childbirth, and the puerperium (CCS11), congenital anomalies (CCS14), and certain conditions originating in the perinatal period (CCS15). The third group is the in-betweens (having mixed signs of *NTDCI*), comprised of diseases of the blood and blood-forming organs (CCS4), mental illness (CCS5), diseases of the nervous system and sense organs (CCS6), diseases of the respiratory system (CCS8), diseases of the digestive system (CCS9), diseases of the genitourinary system (CCS10), diseases of the skin and subcutaneous tissue (CCS12), injury and poisoning (CCS16), symptoms, signs, and ill-defined conditions and factors influencing health status (CCS17), and residual codes unclassified diseases (CCS18).

The complete bidirectional relationship between the healthcare expenditures of disease *i* and disease *j* (measured by the *NPDCI_ij_*) is represented in Figure 1. As indicated in Figure 1a, there are 153 (=$C_{2}^{18}$) net pairwise relationships among these eighteen disease-specific healthcare expenditures. The *TCI* is 87.20%, suggesting that approximately 87.20% of the total forecast error variance can be explained by spillovers from shocks to these eighteen disease-specific healthcare expenditures. In order to better understand the complete network relationship among these eighteen disease-specific healthcare expenditures, we further decomposed the complete connectedness network into the connectedness network of net transmitters (average *NTDCI* > 0, see Figure 1b) and net receivers (average *NTDCI* < 0, see Figure 1c). It is evident that the overall magnitude of transmission or reception of spillovers (indicated by the size of nodes) for net transmitters is much higher than that for net receivers, and in general, the strength of spillovers (shown by the thickness of arrows) between a pair of CCS codes within the connectedness network of net transmitters is much higher than that within the connectedness network of net receivers. Figure 2 reveals the dynamic connectedness network for the eighteen disease-specific healthcare expenditures. For the purpose of our discussion, we could further separate these eighteen disease-specific healthcare expenditures into three groups from Figure 2: the pure net transmitters of spillover (all having positive signs of *NTDCI*), pure net receivers of spillover (all with negative signs of *NTDCI*), and in-betweens (having mixed signs of *NTDCI*) groups.

Table 1 presents the network transmission mechanisms of disease-specific and total healthcare expenditure spillovers. Since three influence statistics, RStudent, DFFITS (Difference in Fits), and CovRatio statistics identified 7–26 (or 3.7% to ~13.76%) outliers, the application of the OLS (Ordinary Least Square) method in the regression analyses would bias the statistical inference. Hence, we employed the RLS regression with the MM-estimation (Modified Maximum likelihood-type estimation) suggested by Yohai [22] to analyze the determinants of the disease-specific and total healthcare expenditure spillovers. Note that population ageing, in general, resulted in an increase in the elderly population (aged 65 and above) with a synchronized decrease in the young population (aged 15 and below). Because of this, the young-age and old-age economic dependency ratios diverge in opposite directions. If the effects of cyclical components of the young age and old age economic dependency ratios on cyclical components of the *NTDCI* and *TCI* are negative (positive) and positive (negative), respectively, we said that demographic transition has a convex (concave) effect on disease-specific and total healthcare expenditure spillovers, meaning that these spillovers first decrease (increase) as the young population grows, and then increase (decrease) as the elderly population expands.

As shown in Table 1, we found the cyclical component of the old-age economic dependency ratio has a significantly positive effect on the cyclical component of the *TCI*, but the cyclical component of the young-age economic dependency ratio has an insignificant effect on it. This result means that the deviation from the long-run trend of the old-age economic dependency ratio is positively associated with the deviation from the long-run trend of the total healthcare expenditure spillover. However, any deviation from the long-run trend of the young-age economic dependency ratio is unrelated with that from the long-run trend of the total healthcare expenditure spillover. The same condition has been found in the relationship between the *NTDCI* of neoplasms (CCS2) and the demographic variables used for our analyses.

In addition, the significantly convex effects of demographic transition on disease-specific spillovers were found in some clinical diagnosis-related groups of diseases, these being infectious and parasitic diseases (CCS1), endocrine, nutritional, and metabolic diseases and immunity disorders (CCS3), mental illness (CCS5), diseases of the circulatory system (CCS7), diseases of the musculoskeletal system and connective tissue (CCS13), and congenital anomalies (CCS14). Significantly concave effects of demographic transition on disease-specific spillovers were found in other clinical diagnosis-related groups of diseases, these being diseases of the respiratory system (CCS8), complications of pregnancy, childbirth, and the puerperium (CCS11), and certain conditions originating in the perinatal period (CCS15).

Moreover, the cyclical components of three disease-specific healthcare expenditure spillovers (those of diseases of the blood and blood-forming organs (CCS4), injury and poisoning (CCS16), symptoms, and residual codes unclassified diseases (CCS18)) are negatively related to cyclical components of the old-age economic dependency ratio, meaning that population ageing may offset the spillover effects from these three clinical diagnosis-related groups of diseases. These negative relationships are likely to result from either increasingly efficient treatment for these diseases or increasingly accurate diagnoses for these diseases as the morbidities expand. Demographic transition did not generate any significant results on healthcare expenditure spillovers from some clinical diagnosis-related groups of diseases, these being diseases of the nervous system and sense organs (CCS6), diseases of the digestive system (CCS9), and ill-defined conditions and factors influencing health status (CCS17).

The estimated coefficients of the cyclical components of the composite leading index are significantly positive for the *TCI* and *NTDCI* of three clinical diagnosis-related groups of diseases, these being neoplasms (CCS2), diseases of the blood and blood-forming organs (CCS4), and diseases of the musculoskeletal system and connective tissue (CCS13). However, they are significantly negative for the *NTDCI* of four clinical diagnosis-related groups of diseases, these being endocrine, nutritional, and metabolic diseases and immunity disorders (CCS3), mental illness (CCS5), diseases of the skin and subcutaneous tissue (CCS12), and residual codes unclassified diseases (CCS18). Despite the significantly negative relationship between the *NTDCI* and medical price index that was found in the case of diseases of the genitourinary system (CCS10), there exists a positive relationship between the *NTDCI* and medical price in five of the eighteen clinical diagnosis-related groups of diseases (namely, endocrine, nutritional, and metabolic diseases and immunity disorders (CCS3), mental illness (CCS5), diseases of the nervous system and sense organs (CCS6), diseases of the circulatory system (CCS7), and diseases of the digestive system (CCS9)).

The impact of the volume of primary care utilization on the *NTDCI* was found to be significantly negative for one-third of the eighteen clinical diagnosis-related groups of diseases (i.e., infectious and parasitic diseases (CCS1), neoplasms (CCS2), endocrine, nutritional, and metabolic diseases and immunity disorders (CCS3), diseases of the circulatory system (CCS7), diseases of the musculoskeletal system and connective tissue (CCS13), and ill-defined conditions and factors influencing health status (CCS17)). The impact was significantly positive for four of the eighteen clinical diagnosis-related groups of diseases (namely, diseases of the genitourinary system (CCS10), complications of pregnancy, childbirth, and the puerperium (CCS11), congenital anomalies (CCS14), and certain conditions originating in the perinatal period (CCS15)). It was found to be insignificant for the remaining eight clinical diagnosis-related groups of diseases. It is possible that the negative effects of the volume of primary care utilization on the *NTDCI* dominate the positive and insignificant effects, leading to a significant and negative effect of volume of primary care utilization on the *TCI*.

Although a positive effect of Baumol’s cost disease (defined as a gap between the change of wage in the healthcare sector and the growth of productivity of the whole economy) on healthcare expenditure growth is predicted by Baumol’s unbalanced growth model [18], the relationships between the Baumol’s cost disease and healthcare expenditure spillovers are identified as positive for one-third of the eighteen clinical diagnosis-related groups of diseases (i.e., infectious and parasitic diseases (CCS1), neoplasms (CCS2), diseases of the blood and blood-forming organs (CCS4), diseases of the musculoskeletal system and connective tissue (CCS13), congenital anomalies (CCS14), and symptoms, signs, and ill-defined conditions and factors influencing health status (CCS17)), ambiguous for seven of eighteen clinical diagnosis-related groups of diseases (namely, endocrine, nutritional, and metabolic diseases and immunity disorders (CCS3), diseases of the respiratory system (CCS8), complications of pregnancy, childbirth, and the puerperium (CCS11), diseases of the skin and subcutaneous tissue (CCS12), diseases of the musculoskeletal system and connective tissue (CCS13), congenital anomalies (CCS14), certain conditions originating in the perinatal period (CCS15), injury and poisoning (CCS16), and residual codes unclassified diseases (CCS18)), and even negative for five of eighteen clinical diagnosis-related groups of diseases (viz. mental illness (CCS5), diseases of the nervous system and sense organs (CCS6), diseases of the circulatory system (CCS7), diseases of the digestive system (CCS9), and diseases of the genitourinary system (CCS10)). These results may lead to an insignificant effect of Baumol’s cost disease on total healthcare expenditure spillovers.

## 4. Discussion

The underlying logic for using the sophisticatedly dynamic connectedness network analyses for this study is twofold: first, this methodology is capable of elucidating the network relationships among disease-specific healthcare expenditure spillovers and preventing biases from structural change and endogeneity of different disease-specific healthcare expenditures. Second, the establishment of network relationships among disease-specific healthcare expenditure spillovers allowed us to unravel the black box of healthcare expenditure growth processes. Since spillovers from shocks to the eighteen disease-specific healthcare expenditures (based on the multi-level CCS categories) explained a substantial proportion (approximately 87.20%) of the total forecast error variance during our study period of January 2000 to ~September 2015 (see *TCI* in Figure 1), we are able to disentangle the healthcare expenditure growth processes black box through the newly developed dynamic connectedness network analyses. There are several policy implications that can be drawn from our empirical results.

First, the evidence generated from the RLS estimates shown in Table 1 suggests that population ageing is one of the main forces driving the diffusion of total healthcare expenditure. This result reflects the fact that the rapidly increasing rate of population ageing in Taiwan expands demand for healthcare services. Our results are consistent with those from previous studies on the determinants of healthcare expenditure growth [1,2,3,4,5,6,7]. Although demographic transition is an important determinant of total healthcare expenditure spillover, the effect of demographic transition on individual disease-specific healthcare expenditure spillovers is variant, based on the results from Table 1. Specifically, the net effects of population ageing (in terms of the sum of the estimated coefficients of young-age and old-age economic dependency ratio) on disease-specific healthcare expenditure spillovers from pure net transmitters of spillover (including the infectious and parasitic diseases (CCS1), neoplasms (CCS2), endocrine, nutritional, and metabolic diseases and immunity disorders (CCS3), diseases of the circulatory system (CCS7), and diseases of the musculoskeletal system and connective tissue (CCS13)) are positive, but those from some net receivers of spillover (such as complications of pregnancy, childbirth, and the puerperium (CCS11) and certain conditions originating in the perinatal period (CCS15)) are negative.

Consequently, the increase in healthcare expenditures resulting from pure net transmitters of spillover will drive a further increase in all other disease-specific healthcare expenditures within the network, and this transmission effect is mostly likely to be amplified as Taiwan’s elderly population persistently grows. Contrarily, changes in healthcare expenditure resulting from pure net receivers of spillover (mostly related to diseases of pregnancy, childbirth, and the puerperium in the perinatal period) are driven by the changes in all other disease-specific healthcare expenditures within the network, and this transmission effect is likely to grow stronger as Taiwan’s total fertility rate continuously decreases. In order to counter-balance a dramatic upward trend in total healthcare expenditure, the priority among health promotion policies should be those policies that target preventive intervention for those diseases classified as pure net transmitters of spillover, and provide sufficient incentives for residents in Taiwan to increase the total fertility rate.

Second, Taiwan’s NHI is a compulsory program with universal coverage on inpatient, outpatient, dental, emergency department care services, and traditional Chinese medicine. A household of four members only pays US $100, approximately 2% of household income [35]. As a result, Taiwan’s healthcare market is highly regulated with regards to the price of healthcare services, and in turn, the wage level in the healthcare sector is more stable than that in other sectors. Although the medical price and Baumol’s cost diseases (measuring the unbalanced growth of productivities between the healthcare sector and the whole economy) have some impacts on some disease-specific healthcare expenditure spillovers, these two macroeconomic variables have little impact on the interconnectedness of these eighteen disease-specific healthcare expenditures, a finding consistent with a recent investigation on the effect of Baumol’s cost diseases on healthcare expenditure in the OECD countries [16].

Third, it is worth addressing that both the beneficiaries and healthcare providers of Taiwan’s NHI program have incentives to utilize or provide more healthcare services in hospitals due to the following characteristics of Taiwan’s healthcare system: (a) Taiwan’s NHI system lacks of a compulsory referral mechanism, (b) out-of-pocket payments to access hospital outpatient care is quite low and the cost per outpatient visit ranges from NT $180 (or US $6) in district hospitals to NT $570 (or US $19) in medical centers, and (c) reimbursement payments for outpatient care provided in hospitals are 2 to ~4 times higher than those for local clinics [36]. Thus, our results shown in Table 1, identifying a negative relationship between primary care utilization and total healthcare expenditure spillover, imply that the promotion of primary care utilization could serve as a policy instrument to mitigate total healthcare expenditure growth under Taiwan’s NHI system. Particularly, primary care is predicted to effectively reduce diffusion of healthcare expenditure in pure net transmitters of spillover and in the clinical diagnosis-related group of symptoms, signs, and ill-defined conditions and factors influencing health status (CCS17), but it should significantly increase diffusion of healthcare expenditure in pure net receivers of spillover and in the clinical diagnosis-related group of injury and poisoning (CCS16).

Fourth, the relationship between business cycles and healthcare expenditure is associated with the contradictory income and substitution effects on individuals’ health behaviors. The income effect refers to a change in the individual’s ability to afford healthcare services and other goods and services, while the substitution effect refers to an adjustment in the opportunity cost of health-promoting activity relative to work [37]. If the income effect is higher (lower) than the substitution effect, healthcare expenditure will be pro-cyclical (counter-cyclical) with respect to business cycles. With this backdrop, either a pro-cyclical pattern (such as for the *NTDCI* of neoplasms (CCS2), diseases of the blood and blood-forming organs (CCS4), and diseases of the musculoskeletal system and connective tissue (CCS13)) or a counter-cyclical pattern (such as for the *NTDCI* of endocrine, nutritional, and metabolic diseases and immunity disorders (CCS3), mental illness (CCS5), diseases of the skin and subcutaneous tissue (CCS12), and residual codes unclassified diseases (CCS18)) in healthcare expenditure spillovers with respect to business cycles were found in different disease-specific healthcare expenditures. Despite this, economic booms increased the interconnectedness of the eighteen disease-specific healthcare expenditures, a result which reflects the pro-cyclical pattern in mortality or morbidity with respect to business cycles in the short-run [37,38,39]. It follows that it is reasonable for policymakers to provide stronger health interventions for neoplasms (CCS2), diseases of the blood and blood-forming organs (CCS4), and diseases of the musculoskeletal system and connective tissue (CCS13) in economic boom periods than that in recession periods. Appropriate targets for preventative intervention during recessions would be the endocrine, nutritional, and metabolic diseases and immunity disorders (CCS3), mental illness (CCS5), diseases of the skin and subcutaneous tissue (CCS12), and residual codes unclassified diseases (CCS18).

Finally, the limitations of this study should be addressed: First, weekly healthcare expenditures were collected for the connectedness network analyses, because the sample size of the high frequency data was sufficient to estimate 153 (=$C_{2}^{18}$) bidirectional relationships between two disease-specific healthcare expenditures. However, the explanatory variables used to analyze the determinants of healthcare expenditure spillovers are reported monthly. In order to proceed with our analyses, healthcare expenditure spillovers were aggregated into monthly data. Such temporal aggregation results in aggregation bias unless the homothetic assumption is imposed on the utility or production functions [40]. The tests for homothetic assumption on consumer preference or production technology are beyond the scope of this study. Second, cyclical components were used for our RLS regression analyses, so inferences generated from this study are restricted regarding the short-run effect of determinants of healthcare expenditure spillovers. Third, the dependent variables of the RLS regression analyses are disease-specific healthcare expenditure spillovers (measured by the connectedness indices) rather than disease-specific healthcare expenditures per se, so this study is not capable of forecasting future healthcare expenditures that would give specific solutions regarding whether disease-specific or generic healthcare expenditures are optimal for unforeseen health risks, such as endemics/pandemics. We left this topic for future research. Finally, since healthcare expenditure (or utilization) and demographic data are aggregated time series data, in order to avoid the ecological fallacy of research, our empirical results neither refer to an individual’s decision in seeking care nor the change of an individual’s behavior at his/her specific age in response to changes in healthcare utilization.

## 5. Conclusions

The significance of this study is to examine factors influencing the network transmission mechanisms underlying the determinants of healthcare expenditure growth through the dynamic connectedness network and the RLS regression analyses for the first time. Our results show that demographic transition and business cycles are key factors increasing the interconnectedness of different disease-specific healthcare expenditures, while the promotion of primary care utilization could reduce total healthcare expenditure spillover. In order to reduce diffusion of disease-specific healthcare expenditures, health promotion activities should focus on those clinical diagnosis-related groups of diseases classified as pure net transmitters of spillover, and preventive interventions targeting different diseases should be activated in different phrases of the business cycle.

## Figures and Tables

**Table 1:** Robust Least Square Estimates for Total Connectedness and Net Total Directional Connectedness Indices.

Connectedness Indices	Young-Age Economic Dependency Ratio		Old-Age Economic Dependency Ratio		Composite Leading Indicator		Medical Price Index		Volume of Primary Care		Growth of Baumol’s Cost (×10^−2^)		Outliers Detected from OLS		
	Coef	(Z-Stat)	Coef	(Z-Stat)	Coeff	(Z-Stat)	Coeff	(Z-Stat)	Coef	(Z-Stat)	Coeff	(Z-Stat)	RStudent	DFFFITS	CovRatio
*TCI*	−0.515	(−1.30)	2.853	(4.22) ***	2.495	(2.19) **	−0.512	(−0.17)	−0.041	(−2.02) **	0.380	(0.25)	7	6	21
*CCS1*	−0.389	(−1.76) *	0.634	(1.68) *	−1.016	(−1.59)	−0.379	(−0.23)	−0.026	(−2.27) **	1.682	(1.98) **	7	8	21
*CCS2*	−0.216	(−0.86)	1.313	(3.06) ***	1.735	(2.39) **	−2.035	(−1.07)	−0.034	(−2.64) ***	2.002	(2.07) **	9	12	16
*CCS3*	−0.585	(−4.33) ***	1.261	(5.47) ***	−0.792	(−2.03) **	2.068	(2.03) **	−0.028	(−4.11) ***	−0.002	(0.00)	9	11	22
*CCS4*	0.128	(0.69)	−0.627	(−1.98) **	0.957	(1.79) *	2.129	(1.52)	−0.007	(−0.71)	2.328	(3.26) ***	8	7	18
*CCS5*	−0.180	(−1.65) *	0.339	(1.82) *	−1.078	(−3.42) ***	5.413	(6.56) ***	0.007	(1.19)	−0.775	(−1.85) *	12	13	22
*CCS6*	0.018	(0.16)	−0.133	(−0.69)	0.025	(0.08)	2.641	(3.08) ***	0.005	(0.91)	−1.416	(−3.24) ***	9	16	17
*CCS7*	−0.619	(−4.42) ***	1.615	(6.76) ***	−0.415	(−1.03)	3.231	(3.06) ***	−0.032	(−4.42) ***	−1.101	(−2.05) **	9	16	17
*CCS8*	1.250	(3.47) ***	−2.660	(−4.33) ***	−1.674	(−1.61)	−3.909	(−1.44)	0.008	(0.45)	0.147	(0.11)	8	14	16
*CCS9*	−0.085	(−0.61)	0.371	(1.56)	−0.106	(−0.26)	2.318	(2.21) **	−0.007	(−1.02)	−1.506	(−2.82) ***	10	13	12
*CCS10*	−1.019	(−1.74) *	0.803	(0.81)	−1.591	(−0.94)	−9.577	(−2.17) **	0.050	(1.67) *	−5.196	(−2.31) **	10	11	20
*CCS11*	0.429	(3.28) **	−0.774	(−3.47) ***	0.557	(1.48)	0.504	(0.51)	0.015	(2.19) **	−0.457	(−0.91)	12	10	26
*CCS12*	−0.071	(−0.60)	−0.188	(−0.93)	−0.835	(−2.45) **	1.332	(1.49)	0.008	(1.26)	−0.567	(−1.25)	8	9	18
*CCS13*	−0.206	(−2.11) **	0.339	(2.04) **	0.915	(3.25) ***	−0.149	(−0.20)	−0.009	(−1.82) *	0.746	(1.99) **	14	13	21
*CCS14*	−0.265	(−2.33) **	0.413	(2.14) **	0.080	(0.25)	0.633	(0.74)	0.006	(0.98)	0.988	(2.27) **	7	9	22
*CCS15*	0.240	(2.31) **	−0.824	(−4.64) ***	0.149	(0.49)	−0.247	(−0.31)	0.016	(2.97) ***	0.265	(0.66)	6	7	20
*CCS16*	0.090	(0.90)	−0.443	(−2.60) ***	−0.142	(−0.49)	−0.918	(−1.22)	0.010	(1.87) *	−0.448	(−1.17)	7	7	25
*CCS17*	−0.199	(−1.05)	0.045	(0.14)	−0.141	(−0.26)	0.404	(0.28)	−0.017	(−1.79) *	1.810	(2.49) **	6	9	18
*CCS18*	0.011	(0.09)	−0.711	(−3.48) ***	−0.704	(−2.04) **	0.868	(0.96)	0.008	(1.31)	0.511	(1.11)	9	10	15

Note: The MM estimation was used for the Robust Least Square Estimates. ***, **, and * indicate significance at the 1%, 5%, and 10% significance level, respectively. RStudent, DFFITS, and CovRatio represent the studentized residual, the scaled difference in fitted values for that observation between the original equation and an equation estimated without that observation, and the ratio of the determinant of the covariance matrix of the coefficients from the original equation to the determinant of the covariance matrix from an equation without that observation, respectively. *TCI* and *CCSi* (*i* = 1, 2,…, 18) represent total connectedness and net total directional connectedness indices of CCS code *i*, respectively.

## Data Availability

Data for this research came from Taiwan’s National Health Insurance Research Database, NHIRD, available at https://nhird.nhri.org.tw/en/Data_Files.html (accessed on 1 January 2021), the Demographic Statistics Database, DSD, available on https://www.ris.gov.tw/app/portal/346 (accessed on 1 January 2021), the Macroeconomics Statistics Database, MSD, available at https://statdb.dgbas.gov.tw/pxweb/Dialog/statfile9L.asp (accessed on 1 January 2021) and Taiwan Business Indicators Database, TBID, available at https://index.ndc.gov.tw/n/en#/ (accessed on 1 January 2021), administered by the Taiwanese government.

## Supplementary Materials

The Supplementary Materials of this study is available online at https://www.mdpi.com/2227-9032/9/3/319/s1.
