# Supplementary material for: On the Network Transmission Mechanisms of Disease-Specific Healthcare Expenditure Spillovers: Evidence from the Connectedness Network Analyses
Source: Healthcare (Basel). 2021 Mar 13;9(3):319. doi: 10.3390/healthcare9030319 (PMC8001239; doi:10.3390/healthcare9030319)
Supplement: Supplementary file 1 [file healthcare-09-00319-s001.pdf]

healthcare

MDPI

Online Supplementary Materials

1. TVP-VAR-based Connectedness Network Analyses

Specifically, we define the pairwise directional connectedness from disease-specific healthcare expenditure *j* to disease-specific healthcare expenditure *i* as follows:

$$\text{Eq (A1)}\quad \tilde{\varphi}_{ij,t}(h) = \frac{\sum_{t=1}^{h-1}(\psi_{ij,t}^{g})^2}{\sum_{i=1}^{N}\sum_{t=1}^{h-1}(\psi_{ij,t}^{g})^2} \tag{1}$$

Where $\tilde{\varphi}_{ij,t}^{g}(h)$ represents the disease-specific healthcare expenditure *j's* contribution to the disease-specific healthcare expenditure *i's* H-step-ahead generalized forecast error variance decomposition at time *t* = *1, 2,…,N*. $\psi_{ij,t}^{g} = S_{ij,t}^{-0.5} A_{h,t} \sum_t \varepsilon_{ij,t}$, $S_{ij,t}$ and $A_{h,t}$ are parameters matrices under a stationary TVP-VAR(1) process with time-varying volatility as follows: $y_t = \beta_t y_{t-1} + \varepsilon_t$, $\varepsilon_t \sim N(0, S_t)$, $\beta_t = \beta_{t-1} + \nu_t$, $\nu_t \sim N(0, R_t)$, and $y_t = A_t \varepsilon_{t-1} + \varepsilon_t$. $\sum_t$ denotes the covariance matrix for error $\varepsilon_{ij,t}$. We further normalize $\tilde{\varphi}_{ij,t}(h)$ in terms of $\sum_{j=1}^{N}\tilde{\varphi}_{ij,t}^{g}(h) = 1$ and $\sum_{i,j=1}^{N}\tilde{\varphi}_{ij,t}^{N}(h) = N$, and the Total Connectedness Index (*TCI*) representing interconnectedness of the network of all different disease-specific healthcare expenditures is given by:

$$\text{Eq (A2)}\quad C_t^{g}(h) = \frac{\sum_{i,j=1,i\neq j}^{N}\tilde{\varphi}_{ij,t}^{g}(h)}{\sum_{j=1}^{N}\tilde{\varphi}_{ij,t}^{g}(h)} \times 100 \tag{2}$$

Note that *TCI* measures the average contribution of spillovers from shocks to all disease-specific healthcare expenditures to the total forecast error variance. In addition, this flexible specification of equation (A2) allows us to identify the directional spillovers of the disease-specific healthcare expenditure *i* to all others *j* as follows:

$$\text{Eq (A3)}\quad C_{i\to j,t}^{g}(h) = \frac{\sum_{j=1,i\neq j}^{N}\tilde{\varphi}_{ji,t}^{g}(h)}{\sum_{j=1}^{N}\tilde{\varphi}_{ji,t}^{g}(h)} \times 100 \tag{3}$$

Analogously, the directional spillovers of all other disease-specific healthcare expenditures to the disease-specific healthcare expenditure *i* is written by:

$$\text{Eq (A4)}\quad C_{i\leftarrow j,t}^{g}(h) = \frac{\sum_{j=1,i\neq j}^{N}\tilde{\varphi}_{ij,t}^{g}(h)}{\sum_{j=1}^{N}\tilde{\varphi}_{ij,t}^{g}(h)} \times 100 \tag{4}$$

We denoted total directional connectedness to others and from others as $C_{i\to j,t}^{g}(h)$ and $C_{i\leftarrow j,t}^{g}(h)$, respectively. Therefore, the Net Total Directional Connectedness Index (*NTDCI*) is computed as:

$$\text{Eq (A5)}\quad C_{i,t}^{g}(h) = C_{i\to j,t}^{g}(h) - C_{i\leftarrow j,t}^{g}(h) \tag{5}$$

A positive sign for the *NTDCI* ($C_{i,t}^{g}(h)$ > 0) demonstrates one condition, in which disease-specific healthcare expenditure i is driving the network, and a negative sign for the *NTDCI* ($C_{i,t}^{g}(h)$ <0) illustrates the other condition, in which disease-specific healthcare expenditure i is driven by the network. Finally, the net pairwise directional connectedness index (*NPDCI*) can be broken down by the *NTDCI* to evaluate the bidirectional relationship between healthcare expenditures of disease *i* and disease *j* as follows:

$$\text{Eq (A6)}\quad NPDC_{ij}(h) = \frac{\tilde{\varphi}_{ji,t}^{g}(h) - \tilde{\varphi}_{ij,t}^{g}(h)}{N} \times 100 \tag{6}$$

The net pairwise directional connectedness between two different disease-specific healthcare expenditures is the variance of the overall shocks that the disease-specific healthcare expenditure *i* transmitted to the disease-specific healthcare expenditure *j*, and vice versa.

2. Results

2.1. Descriptive Statistics and Unit Root Tests

Table A1 summarizes the descriptive statistics and the PP (Phillips and Perron) unit root tests [41] of weekly aggregate real healthcare expenditures per capita for eighteen clinical diagnosis-related groups of diseases classified by the CCS of the US Agency for Healthcare Research and Quality (AHRQ) [31]. As indicated by Table A1, the mean of weekly expenditure per capita for various clinical diagnosis-related groups of diseases ranges from NT $2.64 (US $0.08) to NT $92.02 (US $3.07). The time plots of these eighteen disease specific healthcare expenditures, shown in Figure A1, illustrate either linear or cyclical trends, and regardless of which demean or de-trend data were used for the PP unit root tests, the null hypotheses of unit root of time series was rejected at 1% significance level. These results validate the application of the TVP-VAR-based connectedness network analyses for the weekly time series of these eighteen disease-specific healthcare expenditures.

Table A1. Descriptive Statistics and Unit Root Tests for Healthcare Expenditure Per Capita.

Clinical Classification Software		Descriptive Statistics (NT $ Constant at 2014)				PP((Phillips and Perron) Unit Root Test	
Code	Description	Mean	SD	Max	Min	Constant	Constant and Trend
CCS1	Infectious and parasitic diseases	36.16	7.40	51.44	14.65	−6.20	−26.99
CCS2	Neoplasms	50.34	14.28	91.59	11.35	−14.22	−28.57
CCS3	Endocrine, nutritional, and metabolic diseases and immunity disorders	61.16	11.61	92.14	23.82	−14.01	−30.73
CCS4	Diseases of the blood and blood-forming organs	14.03	3.12	22.32	6.03	−10.80	−30.44
CCS5	Mental illness	26.80	5.17	61.37	7.91	−26.11	−32.75
CCS6	Diseases of the nervous system and sense organs	40.57	5.00	51.52	14.94	−24.26	−25.12
CCS7	Diseases of the circulatory system	88.84	14.11	127.82	34.73	−18.02	−29.24
CCS8	Diseases of the respiratory system	92.02	11.40	137.76	49.27	−15.05	−15.12
CCS9	Diseases of the digestive system	87.45	9.18	110.61	38.19	−29.73	−28.91
CCS10	Diseases of the genitourinary system	78.41	45.67	202.59	23.15	−37.54	−54.88
CCS11	Complications of pregnancy; childbirth; and the puerperium	6.35	0.77	9.03	3.88	−12.28	−12.46
CCS12	Diseases of the skin and subcutaneous tissue	13.31	1.56	17.29	6.19	−23.79	−27.62
CCS13	Diseases of the musculoskeletal system and connective tissue	39.50	7.39	56.73	9.38	−17.74	−23.72
CCS14	Congenital anomalies	4.53	0.74	6.57	1.55	−23.24	−22.92
CCS15	Certain conditions originating in the perinatal period	2.64	0.44	4.52	1.44	−25.29	−26.95
CCS16	Injury and poisoning	36.61	4.36	72.02	18.84	−19.78	−25.15
CCS17	Symptoms, signs, and ill-defined condi-tions and factors influencing health status	24.36	4.37	34.77	11.25	−8.12	−26.02
CCS18	Residual codes; unclassified	8.41	2.17	13.68	2.25	−10.66	−30.99

Note: Weekly data were collected from 1 January 2000 to 30 September 2015, resulting in a total of 822 weekly observations. US $1 = NT $30 The real healthcare expenditure per capita (constant at 2014) of eighteen clinical diagnosis-related groups of diseases were classified by the multi-level Clinical Classifications Software (CCS) categories from the US Agency for Healthcare Research and Quality were reported.

2.2. Static Connectedness Network Analyses

Table A2 shows the static connectedness network matrix, based on the methodology proposed by Antonakakis and his colleagues [21], for the eighteen disease-specific healthcare expenditures. The ij^{th} element of the matrix shows the estimated contribution to the forecast error variance of the disease-specific healthcare expenditure *i* from shocks to the disease-specific healthcare expenditure *j*, as specified in equation (A1). Accordingly, the off-diagonal sum of elements in each row represents the directional spillovers from all other disease-specific healthcare expenditures to the disease-specific healthcare expenditure *i*, and the off-diagonal sum of elements in each column represents the directional spillovers to all other disease-specific healthcare expenditures from the disease-specific healthcare expenditure *j*, as showed in equations (A3)–(A4). *NTDCI* (Net Total Directional Connectedness Index) is defined as the difference between the sums of each jth column and each ith row (see equation (A5)). The *TCI* (Total Connectedness Index), displayed in the bottom-right corner, is the sum of each column (or row) divided by eighteen, and it is further decomposed by the spillovers from shocks to these eighteen disease-specific healthcare expenditures (see the normalized contribution in the bottom of Table A2). The number of *NPDC* (Net Pairwise Directional Connectedness) transmitters represents the summary of the bidirectional relationship between the healthcare expenditure of disease *i* and that of disease *j*.

Table A2. Static Connectedness Network for 18 Disease-specific Healthcare Expenditures (%)

Clinical Classification System	CCS1	CCS2	CCS3	CCS4	CCS5	CCS6	CCS7	CCS8	CCS9	CCS10	CCS11	CCS12	CCS13	CCS14	CCS15	CCS16	CCS17	CCS18	Contribution from Others
CCS1	11.70	9.60	9.30	7.30	5.30	4.90	8.50	1.70	5.30	2.20	0.70	3.30	7.20	1.50	0.20	5.80	8.80	6.70	88.30
CCS2	9.20	11.10	9.30	7.80	5.80	4.90	8.80	1.50	5.60	3.60	0.40	2.90	7.60	1.70	0.20	5.30	7.70	6.80	88.90
CCS3	9.30	9.10	10.00	6.90	6.30	5.70	9.60	1.70	6.20	2.20	0.50	3.30	7.40	1.60	0.20	5.70	7.40	6.90	90.00
CCS4	10.50	9.00	8.30	9.20	4.40	4.40	7.50	2.00	4.60	5.70	0.70	3.10	7.00	1.90	0.30	5.00	9.00	7.00	90.80
CCS5	8.30	8.00	8.60	6.40	8.70	6.20	8.10	1.90	6.10	2.20	0.90	4.20	7.10	2.10	0.20	6.30	7.00	7.60	91.30
CCS6	7.50	8.50	8.10	6.40	5.90	7.50	8.10	2.20	6.90	2.90	0.90	4.60	8.00	2.50	0.40	6.40	6.90	6.20	92.50
CCS7	8.60	8.80	9.70	6.60	6.20	6.20	10.10	2.40	7.00	1.90	0.50	3.30	7.50	1.70	0.20	5.70	7.00	6.50	89.90
CCS8	4.00	3.80	5.90	3.00	4.20	6.50	8.60	34.00	8.30	3.40	0.80	3.00	3.40	1.40	0.30	3.50	3.20	2.70	66.00
CCS9	6.90	7.80	7.80	6.00	5.80	7.60	8.40	2.90	9.00	2.30	1.10	4.80	8.10	2.50	0.30	6.30	6.70	5.80	91.00
CCS10	3.10	9.80	6.60	5.60	6.40	2.90	6.10	1.40	3.00	38.70	0.10	2.00	3.60	1.20	0.10	2.80	2.60	4.10	61.30
CCS11	6.90	6.20	7.40	4.20	4.90	5.20	8.00	4.10	6.40	5.90	13.70	3.30	5.50	1.90	1.30	4.80	5.60	4.50	86.30
CCS12	7.60	8.60	7.70	6.70	5.70	5.50	7.10	3.30	5.60	5.10	1.40	5.90	6.80	2.30	0.40	6.20	7.50	6.50	94.10
CCS13	7.60	8.70	7.90	6.90	5.60	6.80	7.90	2.10	6.80	2.50	0.80	4.30	8.90	2.40	0.30	6.50	7.40	6.50	91.10
CCS14	8.00	8.80	7.20	6.80	4.50	5.00	6.80	6.50	5.30	4.00	1.20	3.50	6.60	6.40	1.20	5.40	7.10	5.80	93.60
CCS15	5.70	5.80	5.60	4.20	3.30	3.30	5.60	7.00	4.00	9.50	3.90	2.50	4.40	3.70	18.70	3.80	4.90	4.10	81.30
CCS16	8.50	8.80	8.10	6.60	5.80	5.80	7.60	2.50	5.70	2.80	1.00	4.40	7.50	2.30	0.30	8.20	7.40	6.50	91.80
CCS17	9.60	9.00	8.40	7.60	4.90	5.10	7.80	1.70	5.50	2.80	1.10	4.00	7.40	1.70	0.30	5.80	10.50	6.70	89.50
CCS18	10.20	9.20	8.90	7.60	5.60	5.10	7.90	1.90	5.20	2.40	0.90	3.50	7.30	1.90	0.30	5.70	8.50	8.10	91.90
Contribution to others	131.50	139.70	135.00	106.40	90.50	91.10	132.50	47.10	97.70	61.70	16.70	60.00	112.50	34.30	6.40	90.70	114.80	100.90	1569.50
Normalized Contributions	7.31	7.76	7.50	5.91	5.03	5.06	7.36	2.62	5.43	3.43	0.93	3.33	6.25	1.91	0.36	5.04	6.38	5.61	87.20
NET (To-From)	43.20	50.70	45.00	15.60	-0.90	-1.50	42.60	-18.80	6.60	0.40	-69.50	-34.10	21.50	-59.20	-74.80	-1.10	25.30	9.00	*TCI* = 87.20
# of transmitters by *NPDCI*	16.00	16.00	16.00	10.00	6.00	7.00	14.00	4.00	7.00	8.00	1.00	3.00	12.00	2.00	0.00	9.00	12.00	10.00	

Note: The percentage (%) of contribution to the forecast error variance of healthcare expenditure on the Clinical Classification System (CCS) code *i* coming from that on CCS code *j* using the Time-varying Parameters (TVP) VAR model. The row titled "Contribution to others" ("Contribution from others") shows the % of contribution of each CCS code (except the given CCS code) to (from) all others. The net total directional connectedness index (*NTDCI*) is the difference between "Contribution to others" and "Contribution from others" for each CCS code. A positive (negative) sign of the *NTDCI* of CCS *i* suggests that the diseases classified by CCS *i* is a net transmitter (receiver) of healthcare expenditure. The total number of *NPDCI* transmitters by each CCS code *i* is reported in the bottom row of Table A2.

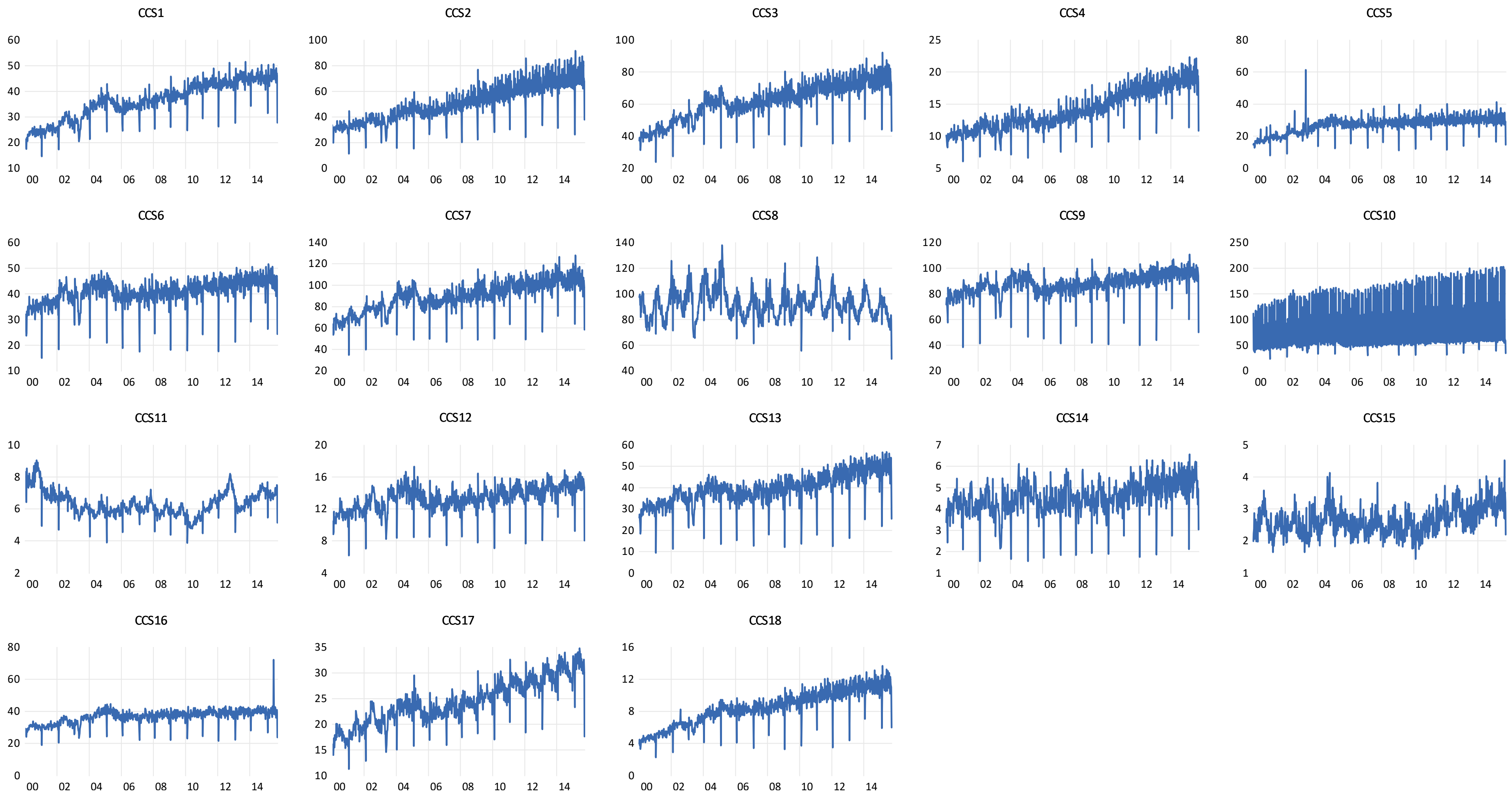

Figure A1. Real Healthcare Expenditure Per Capita (Weekly Aggregate NT $; NT $30 = US $1).

As shown in Table A2, the *TCI* is 87.20%, suggesting approximately 87.20% of the total forecast error variance can be explained by spillovers from shocks to these eighteen disease-specific healthcare expenditures. The highest contributing group (contributing more than 6% to the *TCI*) included neoplasms (CCS2), metabolic diseases and immunity disorders (CCS3), diseases of the circulatory system (CCS7), infectious and parasitic diseases (CCS1), symptoms, signs, and ill-defined conditions and factors influencing health status (CCS17), and diseases of the musculoskeletal system and connective tissue (CCS13). This was followed by the middle contribution group (contributing 4%–6% to the *TCI*) which included diseases of the blood and blood-forming organs (CCS4), residual codes unclassified diseases (CCS18), diseases of the digestive system (CCS9), diseases of the nervous system and sense organs (CCS6), injury and poisoning (CCS16), and mental illness (CCS5). Finally, the group with the lowest contribution (contributing less than 4% to the *TCI*) included diseases of the genitourinary system (CCS10), diseases of the skin and subcutaneous tissue (CCS12), diseases of the respiratory system (CCS8), congenital anomalies (CCS14), complications of pregnancy, childbirth, and the puerperium (CCS11), and certain conditions originating in the perinatal period (CCS15).

The results for net total directional connectedness (NET, seen in the bottom of Table A2) show that ten of these eighteen clinical diagnosis-related groups of diseases (these being infectious and parasitic diseases (CCS1), neoplasms (CCS2), endocrine, nutritional, and metabolic diseases and immunity disorders (CCS3), diseases of the blood and blood-forming organs (CCS4), diseases of the circulatory system (CCS7), diseases of the digestive system (CCS9), diseases of the genitourinary system (CCS10) diseases of the musculoskeletal system and connective tissue (CCS13), symptoms, signs, and ill-defined conditions and factors influencing health status (CCS17), and residual codes unclassified diseases (CCS18)) are net transmitters of spillover. The other eight of these eighteen clinical diagnosis-related groups of diseases (these being mental illness (CCS5), diseases of the nervous system and sense organs (CCS6), diseases of the respiratory system (CCS8), complications of pregnancy, childbirth, and the puerperium (CCS11), diseases of the skin and subcutaneous tissue (CCS12), congenital anomalies (CCS14), certain conditions originating in the perinatal period (CCS15), and injury and poisoning (CCS16)) are net receivers of spillover. The net transmitters of spillover conduct the spillovers of healthcare expenditures through 7–16 clinical diagnosis-related groups of diseases. This is much higher than for the net receivers of spillover (around 0–7 clinical diagnosis-related groups of diseases), as shown in the bottom row of Table A2 (number of transmitters by *NPDCI*).

2.3. Dynamic Connectedness Network Analyses

Since all monthly variables used for the dynamic connectedness network analyses belong to the aggregate time series, we need to deal with the unit root (or non-stationary) property involved in time-series data in order to validate the statistical inference [31]. Prior research into the determinants of healthcare expenditure growth utilized the difference of time series data or cyclical components extracted from time series data to obtain the stationarity of time series data [2–3,8,12,17]. Since healthcare expenditure, demographic variables (such as young-age and old-age economic dependency ratios), composite leading index, medical price index, and primary care utilization are related to business cycles either owing to the definition of the variables or as suggested by evidence from previous studies [13,33], we extracted the cyclic components of these time series data through the Hodrick and Prescott filter method with a smoothing parameter lambda = 14,400 [34]. In addition, since Baumol's cost disease is measured using the growth of the adjusted Baumol cost derived from Colombier [17], the difference of time series data was also used to assure the stationarity of the time series.

Table A3 displays descriptive statistics and unit root tests of *TCI* (Total Connectedness Index), *NTDCI* (Net Total Directional Connectedness Index) and their explanatory variables. As shown in Table A3, either the PP unit root tests with constant or with con-

stant plus trend specifications (or both the PP unit root tests with constant and with constant plus trend specifications) suggest the presence of unit roots in all variables of all levels except for volume of primary care utilization and Baumol's cost disease. Nevertheless, the cyclical components of all variables extracted by the Hodrick and Prescott filter method [34] are stationary time series since both the PP unit root tests with constant and with constant plus trend specifications reject the null hypotheses of unit root of time series at 5% (or rigorous) significance level. Since Baumol's cost disease, constructed based on Colombier [17], has been proved to be a stationary time series, we use the demean series of Baumol's cost disease as consistent with a zero mean of cyclical components of all other variables used for the RLS regression analyses.

Besides, Figure A2 plots the dynamic connectedness network structure of the $NPDCI_{ij}$ among the pure net transmitters of spillover, in-betweens, and pure net receivers of spillover. The accumulated net-pairwise directional relationships are illustrated across three phrases of timespan, separated by two time breaks (i.e., December 2003 and August 2008) of the *TCI*. These time breaks were identified by applying the structural break identification methodology of Bai and Perron [42]. The overall magnitude of transmission or reception of spillovers (indicated by the size of nodes), in general, is highest in the pure net transmitters of spillover, followed by those in the in-between cluster, and is lowest in the pure net receivers of spillover. However, the strength of spillovers (shown by the thickness of arrows) between most pairs of CCS codes (from high to low) is greatest in the in-between cluster, followed by the pure net transmitters of spillover, and is lowest in the pure net receivers of spillover. No matter which group was observed, we found that the overall magnitude of transmission or reception of spillovers is quite stable. However, the strength of spillovers between most pairs of CCS codes expands as our observed timespan extends. As the upward trend of population ageing continues in Taiwan, we expect that demographic transition will play an important role in the determinants of disease-specific healthcare expenditure spillovers.

Table A3. Descriptive Statistics of Total Connectedness, Net Directional Connectedness Indices and their determinants

Panel A: Total Connectedness and Net Total Directional Connectedness Indices													
		Level						Cyclical Components					
Var	Description	Descriptive Statistics				UR Test		Descriptive Statistics				UR Test	
		Mean	SD	Max	Min	Cons	Cons and Trend	Mean	SD	Max	Min	Cons	Cons and Trend
TCI	Total Connectedness Index	87.20	1.46	89.51	84.27	−1.63	−3.59	0.00	0.50	1.22	−1.40	−5.41	−5.40
NTDCI1		2.40	1.24	4.49	0.05	−0.70	−0.92	0.00	0.27	0.70	−0.86	−3.90	−3.89
NTDCI2		2.82	1.31	5.19	0.82	−0.89	−0.50	0.00	0.30	0.72	−0.79	−4.67	−4.66
NTDCI3		2.50	0.97	4.35	0.99	−0.65	−0.83	0.00	0.19	0.56	−0.50	−4.20	−4.19
NTDCI4		0.87	0.88	2.40	−0.65	−1.12	−1.76	0.00	0.23	0.60	−0.46	−3.49	−3.49
NTDCI5		−0.05	0.55	1.03	−0.85	−1.43	−0.35	0.00	0.15	0.43	−0.34	−4.20	−4.19
NTDCI6		−0.08	0.62	0.92	−1.25	−0.83	−0.40	0.00	0.14	0.33	−0.29	−4.67	−4.65
NTDCI7		2.37	0.80	4.09	1.11	−0.68	−0.75	0.00	0.20	0.43	−0.51	−4.12	−4.11
NTDCI8		−1.04	1.05	2.95	−3.16	−2.42	−2.41	0.00	0.56	3.46	−1.31	−4.29	−4.66
NTDCI9	Net Total Directional Connectedness Index of *CCSi*,	0.37	0.32	0.99	−0.30	−1.77	−2.02	0.00	0.17	0.38	−0.44	−4.30	−4.29
NTDCI10	*i* = 1, 2, …, 18	0.02	1.51	5.04	−2.60	−2.38	−3.22	0.00	0.85	4.29	−1.73	−5.14	−5.13
NTDCI11		−3.86	1.71	−0.88	−5.44	0.44	−3.02	0.00	0.19	0.76	−0.48	−5.77	−5.82
NTDCI12		−1.90	1.29	0.21	−4.00	−0.74	−1.25	0.00	0.14	0.37	−0.29	−4.09	−4.08
NTDCI13		1.19	0.45	2.22	0.38	−1.19	−1.69	0.00	0.13	0.36	−0.40	−4.56	−4.55
NTDCI14		−3.29	1.36	−0.34	−4.91	−0.09	−4.50	0.00	0.15	0.80	−0.27	−6.59	−6.58
NTDCI15		−4.16	1.18	−2.11	−5.39	−0.01	−4.23	0.00	0.16	0.82	−0.33	−6.49	−6.49
NTDCI16		−0.06	0.47	0.74	−2.11	−1.66	−2.66	0.00	0.26	0.77	−1.77	−4.61	−4.52
NTDCI17		1.41	0.94	3.07	−0.25	−1.24	−1.94	0.00	0.23	0.59	−0.50	−4.16	−4.15
NTDCI18		0.50	1.09	2.23	−1.30	−0.87	−0.20	0.00	0.16	0.36	−0.34	−4.64	−4.63
**Panel B: Explanatory Variables**													
		**Level**						**Cyclical Components**					
**Var**	**Description**	**Descriptive Statistics**				**UR Test**		**Descriptive Statistics**				**UR Test**	
		**Mean**	**SD**	**Max**	**Min**	**Cons**	**Cons and Trend**	**Mean**	**SD**	**Max**	**Min**	**Cons**	**Cons and Trend**
YEDR	Young-age economic dependency ratio (%)	41.89	7.41	54.14	30.85	−0.93	−0.36	0.00	0.16	0.67	−0.49	−5.52	−5.51
OEDR	Old-age economic dependency ratio (%)	21.54	1.38	24.63	18.92	0.69	−0.94	0.00	0.09	0.22	−0.25	−5.10	−5.10
ln(BLI)	Composite leading indicator (%) in logarithm	4.29	0.23	4.62	3.86	−0.71	−3.00	0.00	0.04	0.07	−0.18	−3.46	−3.45
ln(MPI)	Medical price index (%) in logarithm	4.51	0.10	4.60	4.32	−1.87	−0.98	0.00	0.01	0.03	−0.04	−4.51	−4.49
PCV	Volume of Primary Care (%)	65.40	2.18	70.89	59.81	−5.44	−5.67	0.00	1.82	5.48	−3.98	−6.01	−5.99
BCD	Annual growth of Baumol's Cost (%)	−0.99	3.24	10.08	−17.00	−4.85	−4.83	0.00	3.24	11.08	−16.01	−4.85	−4.83

Note: Monthly total connectedness index and disease-wise net directional connectedness indices were aggregated from weekly data by taking their means, resulting in a total of 189 monthly observations. Old-age (Young-age) economic dependency ratio is the ratio between those aged 65 or above (aged 15 and below) and all people in the labor force. Bold fonts represent 5% (or rigorous) significance levels.

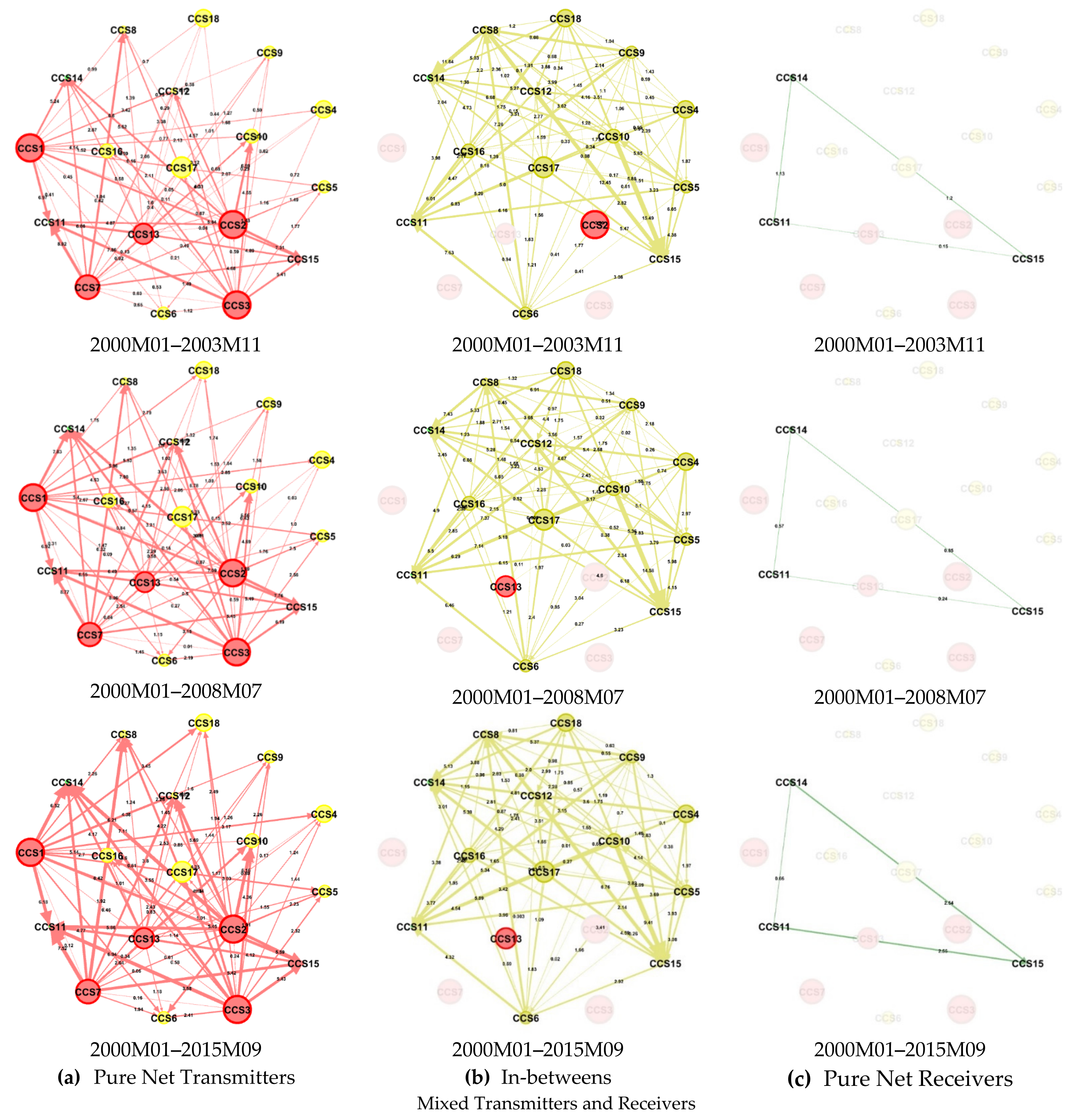

Figure A2. Dynamic Connectedness Network Structure of Net-pairwise Directional Connectedness Indices. Note: The size of nodes indicates the overall magnitude of transmission/reception of spillovers for each Clinical Classification System (CCS) code. The red, yellow, and green colors of each node indicate specific CCS *i* (*i* = 1, 2, ..., 18) that are pure net transmitters, in-betweens, and pure net receivers, respectively. The thickness of the arrows reflects the strength of the spillover between a pair of CCS codes. Thicker arrows indicate stronger spillovers between two CCS codes.

References

1. Liu, Y.M. Population aging, technological innovation, and the growth of health expenditure: Evidence from patients with Type 2 diabetes in Taiwan. *Value Health Reg. Issues* **2020**, *21*, 120–126.
2. Barkat, K.; Sbia, R.; Maouchi, Y. Empirical evidence on the long and short run determinants of health expenditure in the Arab world. *Q. Rev. Econ. Financ.* **2019**, *73*, 78–87.

3. Tian, F.; Gao, J.; Yang, K. A quantile regression approach to panel analysis of health-care expenditure in Organisation for Economic Co-operation and Development countries. *Health Econ.* **2018**, *27*, 1921–1944.
4. Lopreite, M.; Mauro, M. The effects of population ageing on health care expenditure: A Bayesian VAR analysis using data from Italy. *Health Policy* **2017**, *121*, 663–674.
5. Chen, W.Y.; Wen, M.J.; Lin, Y.H.; Liang, Y.W. On the relationship between healthcare expenditure and longevity: Evidence from continuous wavelet analyses. *Qual. Quant.* **2016**, *50*, 1041–1057.
6. Murthy, V.N.R.; Okunade, A.A. Determinants of U.S. health expenditure: Evidence from autoregressive distributed lag (ARDL) approach to cointegration. *Econ. Model.* **2016**, *59*, 67–73.
7. Breyer, F.; Lorenz, N.; Niebel, T. Population ageing and health care expenditures: Is there a Eubie Blake effect? *Eur. J. Health Econ.* **2015**, *16*, 95–112.
8. Bernal-Delgadoa, E.; Comendeiro-Maaløea, M.; Ridao-Lópeza, M.; Rossellób, A.S. Factors underlying the growth of hospital expenditure in Spain in a period of unexpected economic shocks: A dynamic analysis on administrative data. *Health Policy* **2020**, *124*, 389–396.
9. Abdullah, S.M.; Siddiqua, S.; Huque, R. Is health care a necessary or luxury product for Asian countries? An answer using panel approach. *Health Econ. Rev.* **2017**, *7*, 4, doi:10.1186/s13561-017-0144-8.
10. Baltagi, B.; Lagraviness, R.; Moscone, F.; Tosetti, E. Health care expenditure and income: A global perspective. *Health Eco-Nomics* **2017**, *26*, 863–874.
11. You, X.; Okunade, A.A. Income and technology as drivers of Australian healthcare expenditures. *Health Econ.* **2017**, *26*, 853–862.
12. Cleeren, K.; Lamey, L.; Meyer, J.; Ruyter, K.D. How business cycles affect the healthcare sector: A cross-country investigation. *Health Econ.* **2016**, *25*, 787–800.
13. Cinaroglu, S.; Baser, O. The relationship between medical innovation and health expenditure before and after health reform. *Health Policy Technol.* **2018**, *7*, 379–387.
14. Murthy, V.N.R.; Ketenci, N. Is technology still a major driver of health expenditure in the United Sates? evidence from cointegration analysis with multiple structural breaks. *Int. J. Health Econ. Manag.* **2017**, *17*, 29–50.
15. Willemé, P.; Dumont, M. Machines that go 'Ping': Medical technology and health expenditures in OECD countries. *Health Econ.* **2015**, *24*, 1027–1041.
16. Atanda, A.; Menclova, A.K.; Reed, W.R. Is health care infected by Baumol's cost disease? Test of a new model. *Health Econ.* **2018**, *27*, 832–849.
17. Colombier, C. Divers of health-care expenditure: What role does Baumol's cost disease play? *Soc. Sci. Q.* **2017**, *98*, 1603–1621.
18. Baumol, W.J. Health care, education and the cost disease: A looming crisis for public choice. *Public Choice* **1993**, *77*, 17–28.
19. Harris, A.; Sharma, A. Estimating the future health and aged care expenditure in Australia with changes in morbidity. *PLoS ONE* **2018**, *13*, e0201697, doi:10.1371/journal.pone.0201697.
20. Caley, M.; Sidhu, K. Estimating the future healthcare costs of an aging population in the UK: Expansion of morbidity and the need for preventative care. *J. Public Health* **2010**, *33*, 117–122.
21. Antonakakis, N.; Gabauer, D.; Gupta, R.; Plakandaras, V. Dynamic connectedness of uncertainty across developed economies: A time-varying approach. *Econ. Lett.* **2018**, *166*, 63–75.
22. Yohai, V.J. High breakdown-point and high efficiency robust estimates for regression. *Ann. Stat.* **1987**, *15*, 642–656.
23. Linde, S. The formation of physician patient sharing networks in medicare: Exploring the effect of hospital affiliation. *Health Econ.* **2019**, *28*, 1435–1448.
24. DuGoff, E.H.; Fernandes-Taylor, S.; Weissman, G.E.; Huntley, J.H.; Pollack, C.E. A scoping review of patient-sharing network studies using administrative data. *Transl. Behav. Med.* **2018**, *8*, 598–625.
25. Raminani, S.R. International Classification of Diseases. Encyclopedia Britannica. britannica.com. 2014. Available online: https://www.britannica.com/topic/International-Classification-of-Diseases (accessed on 20 April 2020).
26. National Development Council. Population projection for the R.O.C. (Taiwan) 2018-2065. Executive Yuan, Taiwan. 2019. Available online: https://www.ndc.gov.tw/News_Content.aspx?n=114AAE178CD95D4C&sms=DF717169EA26F1A3&s=E1EC042108072B67 (accessed on 20 April 2020).
27. DGBAS (2020). Macroeconomics Statistics Database. Directorate-General of Budget, Accounting and Statistics, Executive Yuan, Taiwan. 2020. Available online: https://statdb.dgbas.gov.tw/pxweb/Dialog/statfile9L.asp (accessed on 20 April 2020).
28. Dangayach, Y.; Gupta, A. Four Asian dragons-evolution and their growth. *Int. J. Adv. Res. Dev.* **2018**, *3*, 158–162.
29. Diebold, F.X.; Yilmaz, K. Measuring financial asset return and volatility spillover, with application to global equity. *Econ. J.* **2009**, *119*, 158–171.
30. Diebold, F.X.; Yilmaz, K. Better to given than receive: Predictive directional measurement of volatility spillovers. *Interna-Tional J. Forecast.* **2012**, *28*, 57–66.
31. AHRQ. Clinical Classifications Software (CCS) 2015. Agency for Healthcare Research and Quality. 2016. Available online: https://www.hcup-us.ahrq.gov/toolssoftware/ccs/CCSUsersGuide.pdf (accessed on 20 April 2020).
32. Enders, W. *Applied Econometric Time Series*; John Wiley&Sons: New York, NY, USA, 2004.
33. Stuckler, D.; Basu, S. *The Body Economics. Why Austerity Kills?* Perseus Book Group: New York, NY, USA, 2013.
34. Hodrick, R.J.; Prescott, E.C.; Postwar, U.S. business cycles: An empirical investigation. *J. Moneycredit Bank.* **1997**, *29*, 1–16.

35. NHIA. 2018–2019 National Health Insurance Annual Report. Taipei: National Health Insurance Administration, Ministry of Health and Welfare. 2019. Available online: http://www.nhi.gov.tw/resource/Webdata/2018-19%E5%85%A8%E6%B0%91%E5%81%A5%E5%BA%B7%E4%BF%9D%E9%9A%AA%E5%B9%B4%E5%A0%B1.pdf (accessed on 20 April 2020).
36. MOHW. National Health Insurance Statistics 2019. Taipei: National Health Insurance Administration, Ministry of Health and Welfare. 2019. Available online: https://dep.mohw.gov.tw/DOS/cp-4648-50662-113.html (accessed on 20 April 2020).
37. Chen, W.Y. Health progress and economic growth in the United States: The continuous wavelet analysis. *Empir. Econ.* **2016**, *50*, 831–855.
38. Liu, Y.H.; Chang, W.S.; Chen, W.Y. Health progress and economic growth in the United States: The Mixed Frequency VAR analyses. *Qual. Quant.* **2019**, *53*, 1895–1911.
39. Ruhm, C.J. Are recessions good for your health? *Q. J. Econ.* **2020**, *115*, 617–650.
40. Varian, H.R. *Microeconomic Analysis*, 3rd ed.; Norton & Company Inc.: New York, NY, USA, 1992.
41. Phillips, P.; Perron, P. Testing for a Unit Root in time series regression. *Biometrika* **1988**, *75*, 335–346.
42. Bai, J.; Perron, P. Multiple structural change models: A simulation analysis. In *Econometric Theory and Practice: Frontier of Analysis and Applied Research-Essays in Honor of Peter Phillips*; Corbae, D., Durlauf, S., Hansen, B.E., Eds.; Cambridge University Press: Boston, MA, USA, 2006; pp. 212–238.
